# 

**Published:** 1856-03

**Authors:** 


### TO BOOK PUBLISHERS.

We have received from Messrs. Samuel S. & William Wood, 261 Pearl Street, New York, Publishers, a number of new medical works, which arrived too late for notice in the present number of the Journal, they will, however, be attended to in our next issue. In reference to the channel through which books may be sent to us, we have to say to those who are interested upon the subject, that for the present, they may be sent, at our expense, by express.

---

### SUBSCRIPTIONS RECEIVED SINCE LAST ISSUE.

C. W. Ashby, Va.; N. D'Alvigny, Ga.; J. F. Moore, Ga.; J. Stone, Ga.; C. S. Haley, Ga.; Dean & Dupree, Ga.; W. T. Grant, Ga.; N. N. Smith, Ga.; Asa W. Griggs, Ga.

### TO CORRESPONDENTS.

☞ All Communications pertaining to the Editorial Department of the Journal, should be addressed to the Editors.

☞ All Communications, having reference to the Subscription, the Advertising or the Financial Department, should be addressed to the Treasurer, J. G. WESTMORELAND, M. D., Dean of the Faculty of the Atlanta Medical College.

---

☞ Messrs. A. TILDEN and W. H. PHILLPOT are authorized Agents for this Journal.

